# War trauma in Homer’s Iliad: a trauma registry perspective

**DOI:** 10.1007/s00068-020-01365-6

**Published:** 2020-04-18

**Authors:** Maria Chicco, Giovanni D. Tebala

**Affiliations:** 1 Department of General Surgery, Wexham Park Hospital, Frimley Health NHS Foundation Trust, Wexham Street, Slough, SL2 4HL UK; 2 Department of Emergency Surgery, John Radcliffe Hospital, Oxford University Hospitals NHS Foundation Trust, Headley Way, Headington, Oxford, OX3 9DU UK

**Keywords:** Trauma, Iliad, Mortality, Injury severity score

## Abstract

**Purpose:**

Homer’s Iliad reports detailed descriptions of war traumas, with precise anatomical references, so that the Iliad can be considered the first trauma registry. We aimed to analyse the Iliad from the perspective of a modern trauma registry: that is, to find historical and local prognostic factors through the epidemiological study of the reported traumas.

**Methods:**

Two different editions of Homer’s Iliad—one in English and one in Italian—were thoroughly studied and epidemiological data were statistically analysed.

**Results:**

148 reports of human traumas were analysed. The majority of traumas (73.6%) involved Trojan warriors, with spears being the most frequent wounding agent (71%). Overall mortality was 84.5% and was higher in the Trojan field (90.8% vs 61.5%). Despite the high mortality, median New Injury Severity Score (NISS) was low, probably due to high prevalence of penetrating mono-systemic trauma. Median NISS was higher in the Trojan group. Compared to the Achaeans, the Trojans had more torso injuries, whereas Achaeans had more injuries to limbs and superficial tissues. However, in both fields, head and neck were more frequently injured.

**Conclusions:**

Homer’s Iliad gives us an interesting insight into war traumas during the siege of Troy. The reported higher mortality within the Trojan army can be explained not only by poetic reasons but also by different military skills.

## Introduction

The Iliad is a Greek epic poem, traditionally attributed to Homer. According to Herodotus, it might have been written in the ninth century BC and describes the Trojan War, probably fought around the twelveth century BC, during the Bronze Age. The war was actually a 10-year siege of Troy (or Ilium) by allied Greek armies. According to Greek mythology, the origins of the Trojan War lie in a banquet held on Mount Olympus for the wedding of Peleus and Thetis, when Eris (the goddess of discord), not invited, tossed a golden apple with ‘to the most beautiful’ inscribed on it. This generated a quarrel between the goddesses Hera, Athena and Aphrodite about which of them was the fairest. To resolve the dispute, Zeus elected the young Trojan shepherd Paris to act as a judge. Each of the three competitors tried to convince Paris with a reward. Hera offered the kingdom of Europe and Asia, Athena offered wisdom and skills and Aphrodite offered Helen of Sparta, the most beautiful of all worldly women, who was married with Sparta’s king Menelaus, brother of Mycenae’s king Agamemnon. Paris awarded the golden apple to Aphrodite and obtained his reward. Menelaus sought help from his brother Agamemnon and other Achaean kings to rescue Helen and they gathered a large army, which besieged Troy for 10 years. The battles were mainly fought outside the walls of Troy.

The historical existence of Troy was put in doubt for many centuries, but the German archaeologist Heinrich Schliemann in 1868 found the remnants of what is now considered the mythological Troy, in present-day Turkey. The excavations found several different layers of buildings and one of them shows signs consistent with the alleged fire started by the Achaeans when they conquered and destroyed Troy. The most accredited version of facts is that the Trojan War was the outcome of a longstanding fight between Achaeans and Trojans for the control of the naval routes to the Black Sea, with Troy being built in an advantageous position on the Turkish shores of Hellespont.

There is no real evidence that the Trojan War was a real one-stage conflict, but at the same time, there is no counter evidence. Some authors state that Homer just collected tales of sporadic and recurrent sieges and attacks of Troy by Achaeans in the Bronze Age and other propose that the Trojan War was a specific conflict. Despite not being professional historians or archaeologists ourselves, we tend to favour this second hypothesis mostly on the basis that different aspects of the Trojan War have been narrated also by other ancient Greek authors. In the Iliad, battles and duels are narrated in abundant detail. In particular, wounds and injuries are described quite thoroughly, so that some authors wondered if Homer himself had a medical background [1]. Mostly, these outstanding descriptions may demonstrate that Homer witnessed several fights and had a deep knowledge of war and its consequences. Several authors described the different traumas in the Iliad [2, 3], which has been considered the oldest wartime surgical report [4].

The recent epidemiological approach to war or civilian traumatic injuries occurs through the implementation of trauma registries. These are useful tools to monitor the epidemiology, pathways, protocols and outcomes of trauma to identify modifiable risk factors for primary and secondary trauma morbidity and mortality. Trauma registries require data to be standardised, periodically analysed and benchmarked on an international basis [5].

In this work, we analyse the Trojan War injuries from a trauma registry perspective to study the epidemiology of war trauma during that conflict and, where possible, to identify the factors leading to the ultimate outcome of the war.

## Materials and methods

Homer’s Iliad in the English translation by Martin Hammond [6] and in the Italian translation by Vincenzo Monti [7] were thoroughly read and analysed. Data for each case of trauma were retrieved and stored in an electronic database (Microsoft Excel for Mac, v. 16.32). Reported injuries of gods and goddesses were excluded. The New Injury Severity Score (NISS) score was calculated for each case as the sum of the squares of the Abbreviated Injury Scale (AIS-98) scores of the three most severe injuries. For the majority of injuries, data were sufficient to understand the underlying trauma mechanism; however, in a few cases, this had to be speculated on the basis of the poetic setting. Data were analysed with GNU PSPP v.1.2.0 (general public license) for Mac and with StatPlus Mac Pro v. 7.1.0.0 for Mac. Categorical data were compared with the Pearson’s Chi-square test. Numerical data were first analysed for distribution and skewness and then compared with parametric (Student’s *t* test) or non-parametric tests (Mann–Whitney *U* test). All the considered variables were taken into account into a binomial logistic regression analysis so as to identify independent ‘prognostic’ factors.

## Results

We identified 148 human traumas described in the Iliad (Table 1). The majority (109/148—73.6%) involved Trojans, whereas only 39/148 (26.3%) involved Achaeans. The weapon most frequently used was the spear (105/148—71.0%). It is interesting to highlight that 72.5% of Trojan wounds were due to spears, in comparison to 66.7% of Achaeans wounds. To the contrary, arrow injuries were much more frequent in Achaeans than in Trojans (17.9% vs 4.6%) ($\upchi$^2^ = 15.34; *p* = 0.009). Blunt, penetrating and multisystem injuries were equally distributed between the two groups.

**Table 1:** Analysis of data

	Total	Achaeans	Trojans	*p*
No. of injuries	148	39 (26.3%)	109 (73.6%)	
Agent				
Spear	105 (71.0%)	26 (66.7%)	79 (72.5%)	0.009
Sword	15 (10.1%)	1 (2.6%)	14 (12.8%)	
Arrow	12 (8.1%)	7 (17.9%)	5 (4.6%)	
Rock	8 (5.4%)	2 (5.1%)	6 (5.5%)	
Multiple	6 (4.1%)	1 (2.6%)	5 (4.6%)	
Other	2 (1.4%)	2 (5.1%)	0	
Mechanism				
Blunt	12 (8.1%)	5 (12.8%)	7 (6.4%)	0.406
Penetrating	130 (87.8%)	32 (82.0%)	98 (89.9%)	
Multiple	6 (4.1%)	2 (5.1%)	4 (3.7%)	
NISS				
	25	16	25	< 0.001
	1–75	1–75	1–75	
NISS ranks				
Mild < 15	22 (15.2%)	15 (39.5%)	7 (6.5%)	< 0.001
Moderate 15–25	89 (61.4%)	18 (47.4%)	71 (66.4%)	
Severe > 25	34 (23.4%)	5 (13.1%)	29 (27.1%)	
Outcome				
Fatal	123 (83.1%)	24 (61.5%)	99 (90.8%)	< 0.001
Non-fatal	23 (15.5%)	15 (38.5%)	8 (7.3%)	
Missing	2 (1.4%)	0	2 (1.8%)	
Body system				
External	2 (1.4%)	2 (5.1%)	0	0.018
Limbs	31 (20.9%)	14 (35.9%)	17 (15.6%)	
Head–Neck	40 (27.0%)	10 (25.6%)	30 (27.5%)	
Chest	24 (16.2%)	4 (10.3%)	20 (18.3%)	
Abdomen	24 (16.2%)	3 (7.7%)	21 (19.3%)	
Multiple	24 (16.2%)	5 (12.8%)	19 (17.4%)	
Unknown	3 (3.0%)	1 (2.6%)	2 (1.8%)	

Data represent number of cases and percentage but for NISS they represent median and range

**Fig. 1:**
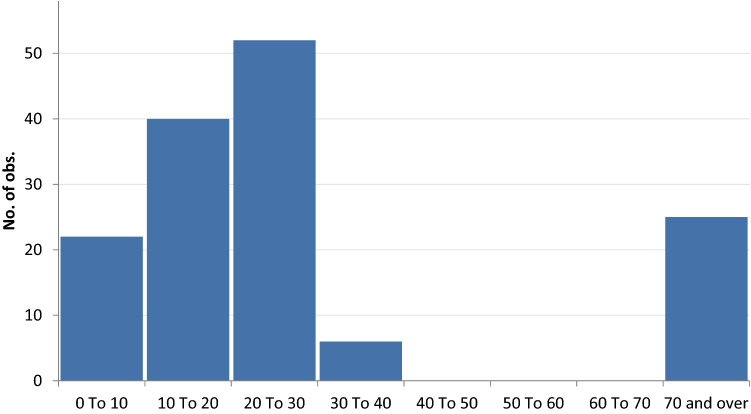
Distribution of NISS score

The vast majority of injuries were fatal and overall mortality for trauma was 84.5% (125/148). Death was immediate, on the scene, in most cases. Mortality was much higher among Trojans than in the Achaean group (90.8 vs 61.5%). NISS could be calculated for 145 cases, while in three cases, there was no information on the mechanism of injury. NISS values were skewed to the right (the right tail is longer than the left one—skewness 1.26) (Fig. 1). Comparison performed with a non-parametric test showed that NISS was higher in the Trojan field (median 25 vs 16, *p* < 0.001). The stratification of NISS into mild, moderate and severe showed a higher prevalence of moderate and severe NISS in the Trojan army with respect to Achaeans (*p* < 0.001).

Injuries to head and neck were the most frequent trauma in the overall series (27.0%), but Achaeans were more frequently hit in the limbs (35.9%) as compared to head and neck (25.6%). Compared to the Achaeans, the Trojans had more chest and abdominal injuries (10.3% vs 18.3% and 7.7% vs 19.3%, respectively). The distribution of wounds was significantly different between the two fields (*p* = 0.018). As expected, most traumas were penetrating (87.8%) and were associated with higher mortality as compared to blunt trauma (86.9% vs 66.7%, *p* = 0.026).

Univariate analysis showed that field (Achaeans vs Trojans), weapon, mechanism of injury, NISS rank and body system involved were all significantly associated with the risk of death (Table 2).

**Table 2:** Univariate analysis (Chi-square test)

	Total	Mortality^a^	Survival^a^	*p*
Field				
Achaeans	39 (26.4%)	24 (61.5%)	15 (38.5%)	< 0.001
Trojans	109 (73.6%)	101 (92.7%)	8 (7.3%)	
Agent				
Spear	105 (70.9%)	93 (88.6%)	12 (11.4%)	0.001
Sword	15 (10.1%)	15 (100%)	0	
Arrow	12 (8.1%)	6 (50%)	6 (50%)	
Rock	8 (5.4%)	5 (62.5%)	3 (37.5%)	
Multiple/other	8 (5.4%)	6 (75%)	2 (25%)	
Mechanism				
Blunt	12 (8.1%)	6 (50%)	6 (50%)	0.002
Penetrating	130 (87.8%)	113 (86.9%)	17 (13.1%)	
Multiple	6 (4.1%)	6 (100%)	0	
NISS ranks				
Mild < 15	22 (15.2%)	0	22 (100%)	
Moderate 15–25	89 (61.4%)	88 (98.9%)	1 (1.1%)	< 0.001
Severe > 25	34 (23.4%)	34 (100%)	0	
Body system				
External	2 (1.4%)	0	2 (100%)	< 0.001
Limbs	31 (20.9%)	16 (51.6%)	15 (48.4%)	
Head–Neck	40 (27%)	37 (92.5%)	3 (7.5%)	
Chest	24 (16.2%)	23 (95.8%)	1 (4.2%)	
Abdomen	24 (16.2%)	24 (100%)	0	
Multiple	24 (16.2%)	22 (91.7%)	2 (8.3%)	
Unknown	3 (2%)	3 (100%)	0	

^a^Percentages within row

Binomial logistic regression analysis for mortality (vs survival) showed that only the variable “field” was independently related to the death outcome (Table 3). Belonging to the Achaean side must be considered a protective factor (negative beta coefficient and odds-ratio < 1).

**Table 3:** Multivariate analysis

Factor	Coefficient	Odds ratio	*p*
Field	− 2.07	0.13	< 0.001
Achaeans (vs Trojans)			
Intercept	− 0.47		

Binomial logistic regression—dependent factor: mortality

Overall model test: $\upchi$^2^ = 18.7, *p* < 0.001

## Discussion

The Trojan War as described by Homer was a peculiar kind of fight. Allegedly, the Achaean allied armies attacked the fortified city of Troy, but the actual fight was not a proper siege business. In fact, the vast majority of fights and duels happened on open fields outside the walls of Troy and the Trojan army was not fully employed to defend the town walls. Moreover, until the use of the Trojan horse, no significant attack was launched against the town itself, probably due to the lack of effective siege engines.

The type of war reported in the Iliad is the so-called “champion warfare” or “single combat” or “monomachy”, where the outcome of the battle depended on single duels between selected “champions” on both sides, probably aimed to avoid unnecessary bloodshed in the total lack of military discipline [8]. Monomachy was characteristic of ancient civilisations living around the Mediterranean basin, including Egyptians, Hebrews, Greeks and Romans [8].

The Iliad has long been studied to gain an insight into battle injuries, anatomical knowledge and trauma management in Ancient Greece [2, 3, 9]. In our study, we analysed the Iliad using a modern trauma registry perspective. Trauma registries have been introduced into the clinical practice in the ‘70 [10] with the purpose of improving the quality of trauma systems through the epidemiological study of traumas and their outcome. However, methodology is not yet uniform and different data are collected by different registries. Further obvious differences exist also between civilian and military registries, making comparison difficult if not impossible. Van Dongen et al. identified 203 key variables fundamental for the implementation of an effective military trauma registry [11]. Expectedly, data available from the Iliad—both direct and extrapolated—are nowhere near those needed for a modern trauma registry; other than the names and the affiliation of the opponents, only five variables could be collected for the majority of cases (type of weapon, mechanism of injury, body area, NISS and outcome), but the overall methodology can be applied.

The number of casualties reported in the Iliad (148) is surprisingly low with respect to the length of the war (10 years); however, it should be noted that the Iliad’s action only covers a period of 52 days in the final year of the conflict. It is possible that Homer selected only those involving the most significant heroes on either side, but the detailed description of duel and traumatic injuries may suggest that Homer had observed similar events directly.

Most casualties involved Trojan warriors, probably due to the evident dominance of the Achaean armies prevailing in number, importance and strength; but other factors, including political considerations, might have played a role.

While the overall median NISS is low (25), mortality is very high at 83.1%. This is likely due to the penetrating nature of most injuries, as well as the poor physical protection and limited availability of effective treatment. It should be noted that most mortalities occurred immediately after the injury. The unrealistic frequency of sudden death in the Iliad has previously been noted; this might be regarded as a poetic convention so as to increase the dramatic effect and maintain the rhythm of the narration [3] but may also be the result of Homer ‘cherry-picking’ the most important duels in terms of significant outcome.

As mentioned, mortality was much higher among Trojans than within the Achaean group (90.8% vs 61.5%) and median NISS is higher for Trojans (25 vs 16). Injury distribution varies significantly between the two opposing armies. While in both groups the most common injury type is head and neck trauma, Trojans experienced a higher incidence of chest and abdomen wounds as compared to Achaeans who, on the other hand, suffered a higher incidence of limb injuries. This can be due to different armours and armaments between Achaeans and Trojans. Unfortunately, Homer’s description of armours is not always reliable as an element of fiction seems to be always present. In fact, appearance and strength of heroes in the Iliad are usually symbolically functional to their importance and relevance [3].

Shields were usually long so as to cover the whole body, but small round shields were also used along with greaves to protect the legs. Protective armours including helmets were usually made of bronze, variably mixed with other materials, such as bull’s hide. For instance, Ajax’s shield had several layers of bull’s skin and one layer of bronze. On the other hand, Paris did not wear any armour but only a leopard-skin, probably to signify that he was protected by a powerful goddess.

Many warriors on both sides used a waist-long cuirass made of several bronze plates covering the whole torso but leaving the neck exposed [12]. This can explain the high incidence of neck injuries on both sides. As mentioned, the description of armours may not always be realistic. As an example, although not impossible, the description of Nestor’s shield as being constituted of pure gold, looks quite unlikely. It could be that it was made of very polished and shining bronze, giving the impression of pure gold.

No significant difference between Achaeans and Trojans is described with regard to weapons and armours; this factor, therefore, may not be crucial in determining the difference in mortality. However, NISS is significantly higher for Trojans than for Achaeans, thus highlighting a possible difference in military skills and strategy, if not an unbalanced divine protection.

This leaves the question open: why was the mortality rate significantly higher among Trojans than among Achaeans? One possibility is that in his poem Homer is psychologically ‘siding’ with Achaeans, and rather inclined to describe combats where Achaean heroes survive their wounds, while Trojans more frequently succumb.

We raise the hypothesis that the different mortality rate might be due to many Achaeans being wounded by arrows, whereas Trojans more often succumbed to spears. Homer may be expressing here some sort of moral criticism of Trojan warfare, relying on distant attacks rather than close combat, in contrast to the ‘heroic’ attitude displayed by Achaeans. In fact, Achaeans incurred a fourfold risk of being hit by an arrow than Trojans. To the contrary, almost three quarters of Trojan wounds were due to spear. This difference should not surprise if we consider the usual dynamics of sieges, where defenders tend to avoid body-to-body fight as much as possible and try to repel the attackers from behind the walls of the fortified town.

The skewed distribution of NISS scores towards the left side of the spectrum shows that the vast majority of traumas involved only one system, which is typical of penetrating injuries. Only a few cases had multisystem trauma, and this does not differ between the two fields. The description of multiple traumas may be functional to creating a more dramatic effect but also to demonstrating the anger of the attacker due to personal, more than collective, reasons. The outcome of the duel between Achilles and Hector is paradigmatic.

In contrast to modern war traumas, where explosions account for most lethal casualties [13], penetrating trauma is associated with increased mortality, although this did not reach statistical significance at the multivariate analysis (Table 3). It has been reported that mortality for penetrating war injuries gradually decreased over time. This survey of the Iliad showed that almost 90% of soldiers with penetrating trauma died. Most deaths in Napoleonic wars were still due to penetrating trauma [14]. During WW1, mortality for penetrating abdominal trauma was still very high (66%) but reduced significantly in WW2 (24%) [15], likely due to better immediate care, more than improved protection devices. In fact, while undoubtedly the protection gear of soldiers improved significantly, this progress was paralleled by the introduction of new and more powerful weapons. Similarly, during the Trojan War, quite primitive offensive weapons (spear, arrow, sword) met minimal resistance by bronze and leather shields, helmets and breastplates. The absence of advanced weapons and the recourse to individual duels, more than massive frontal attacks, account for the extremely low incidence of blunt traumas in the Iliad and for their reduced mortality.

Clearly, the site of injury may also be associated with outcome, mortality being highest in combatants with abdominal or chest injuries. In fact, Achaean and Trojan warriors were usually well shielded by a protective armour covering their torso but leaving neck and limbs exposed; for this reason, a penetrating injury to the torso would have been associated with much more kinetic energy compared to injuries of exposed regions. Therefore, abdominal and chest injury were less likely, but often lethal.

In conclusion, the Iliad gives us an interesting insight into war traumas in ancient Greece. The reported higher mortality among Trojans can be explained by poetic and political reasons, but also by different military techniques and skills. Although the Iliad evidences military superiority of the Achaean army over the Trojans, it is for the historian to understand why the final victory of the Achaeans could be obtained only through Odysseus’ stratagem of the Trojan horse.
