# Correction to: War trauma in Homer’s Iliad: a trauma registry perspective

**DOI:** 10.1007/s00068-020-01393-2

**Published:** 2020-05-29

**Authors:** Maria Chicco, Giovanni D. Tebala

**Affiliations:** 1 Department of General Surgery, Wexham Park Hospital, Frimley Health NHS Foundation Trust, Wexham Street, Slough, SL2 4HL UK; 2 Department of Emergency Surgery, John Radcliffe Hospital, Oxford University Hospitals NHS Foundation Trust, Headley Way, Headington, Oxford, OX3 9DU UK

## Correction to: European Journal of Trauma and Emergency Surgery 10.1007/s00068-020-01365-6

The original version of this article unfortunately contained a mistake. The presentation of Table 1 was incorrect. The corrected Table 1 is given below.

**Table 1:** Analysis of data

	Total	Achaeans	Trojans	*p*
No. of injuries	148	39 (26.3%)	109 (73.6%)	
Agent				
Spear	105 (71.0%)	26 (66.7%)	79 (72.5%)	0.009
Sword	15 (10.1%)	1 (2.6%)	14 (12.8%)	
Arrow	12 (8.1%)	7 (17.9%)	5 (4.6%)	
Rock	8 (5.4%)	2 (5.1%)	6 (5.5%)	
Multiple	6 (4.1%)	1 (2.6%)	5 (4.6%)	
Other	2 (1.4%)	2 (5.1%)	0	
Mechanism				
Blunt	12 (8.1%)	5 (12.8%)	7 (6.4%)	0.406
Penetrating	130 (87.8%)	32 (82.0%)	98 (89.9%)	
Multiple	6 (4.1%)	2 (5.1%)	4 (3.7%)	
NISS				
	25	16	25	< 0.001
	1–75	1–75	1–75	
NISS ranks				
Mild < 15	22 (15.2%)	15 (39.5%)	7 (6.5%)	< 0.001
Moderate 15–25	89 (61.4%)	18 (47.4%)	71 (66.4%)	
Severe > 25	34 (23.4%)	5 (13.1%)	29 (27.1%)	
Outcome				
Fatal	123 (83.1%)	24 (61.5%)	99 (90.8%)	< 0.001
Non-fatal	23 (15.5%)	15 (38.5%)	8 (7.3%)	
Missing	2 (1.4%)	0	2 (1.8%)	
Body system				
External	2 (1.4%)	2 (5.1%)	0	0.018
Limbs	31 (20.9%)	14 (35.9%)	17 (15.6%)	
Head–Neck	40 (27.0%)	10 (25.6%)	30 (27.5%)	
Chest	24 (16.2%)	4 (10.3%)	20 (18.3%)	
Abdomen	24 (16.2%)	3 (7.7%)	21 (19.3%)	
Multiple	24 (16.2%)	5 (12.8%)	19 (17.4%)	
Unknown	3 (3.0%)	1 (2.6%)	2 (1.8%)	

Data represent number of cases and percentage but for NISS they represent median and range

The original article has been corrected.

Data represent number of cases and percentage, but for NISS, they represent median and range.

